# Relationship between Apgar scores and long-term cognitive outcomes in individuals with Down syndrome

**DOI:** 10.1038/s41598-021-90651-3

**Published:** 2021-06-16

**Authors:** Laura del Hoyo Soriano, Tracie C. Rosser, Debra R. Hamilton, Danielle J. Harvey, Leonard Abbeduto, Stephanie L. Sherman

**Affiliations:** 1 Department of Psychiatry and Behavioral Sciences, MIND Institute University of California Davis, Sacramento, CA USA; 2 Department of Human Genetics, Emory University School of Medicine, Atlanta, GA USA

**Keywords:** Genetics, Neuroscience, Psychology, Health care, Medical research, Risk factors, Signs and symptoms

## Abstract

This study examined the contribution of the Apgar score at 1 and 5 min after birth to later cognitive functioning in 168 individuals with Down syndrome who were between 6 and 25 years of age at time of cognitive testing. Our results showed that a lower Apgar score at 1 min was related to a worse performance in later cognitive measures of receptive vocabulary, verbal comprehension and production, visual memory and working memory. Results also showed that a lower Apgar score at 5 min was only related to worse later outcomes of verbal comprehension and production and auditory working memory. Our findings suggest a need for future studies investigating how specific perinatal events reflected in the Apgar score are linked to later cognitive functioning in individuals with Down syndrome.

## Introduction

Down syndrome (DS) is the most common known genetic cause of intellectual disability (ID) and results from an extra copy of all or part of chromosome 21^1^. DS is a complex condition that affects both physical and cognitive development. Although the DS phenotypic features are variable, when compared either to typical developing (TD) controls or to other neurodevelopmental disorders (NDDs), a distinctive cognitive profile is generally observed. This profile is characterized by a general cognitive delay, relative strengths in nonverbal abilities, and impairments beyond mental age expectances in language, phonological processing, verbal memory and verbal working memory^2–6^. However, it is important to note that the cognitive phenotype described above is variable across individuals with DS^7^.

For example, one study reported that standard deviations for implicit memory scores were almost three times larger in a group of individuals with DS compared with a cognitive-level matched group of TD individuals; indeed, some individuals with DS even outperformed TD controls^8^. Several other studies have shown that other cognitive areas, such as executive function, attention, and language are also quite variable across individuals with DS^5,9–12^.

Understanding which factors contribute to the observed within-syndrome variability is crucial, and one of the main challenges to an etiology-specific approach to intervention for those with DS^13^. In this regard, previous investigations have suggested that differences in genetic^14–16^ and environmental factors^17,18^ between individuals with DS are related to the degree of impairment in specific cognitive and behavioral areas. In addition, certain comorbidities associated with trisomy 21 (i.e., congenital heart defects, sleep disorders, low thyroid function) are also thought to contribute to this variability^19–23^. Among the factors contributing to this variability, those occurring in the perinatal period (starting with the 20th or 28th week of gestation through the 1st or 4th week after birth) may be of special importance because this is a period of great vulnerability for the developing brain^24^.

Drawing on studies in the euploid population, the association between perinatal events and long-term cognition is well established^25–35^. In this regard, the Apgar scoring system is an indicator of perinatal adverse events and vulnerabilities^36^. It is based on clinician observation of the newborn’s skin complexion, heart rate, reflex irritability, muscle tone, and respiratory effort, with lower scores reflective of greater problems^37^. The score is typically assigned at 1 min and 5 min after delivery. Studies have suggested that a low Apgar score at 1 min often reflects acute perinatal events compromising oxygen availability at birth^38^ which may influence neurodevelopmental pathways related to cognitive functioning^39^. In addition to delivery complications, a low Apgar score at 5 min has been suggested to reflect events or conditions prior to birth (e.g., abnormalities of gestational length and prenatal growth, congenital malformation)^40^, which may have an impact on neurodevelopment^41,42^ and cognitive function^43^. It is important to note that those events and conditions occurring during pregnancy can lead to acute perinatal events compromising oxygen availability at birth, as well events such as hypoxic-ischemic encephalopathy, hypotony. Consequently, the 1- and 5-min Apgar scores are influenced by overlapping sets of factors and are thus highly correlated^40,44^. Perinatal hypoxia is further thought to have especially serious effects on the hippocampus and prefrontal cortex, which are responsible for several neurocognitive functions including memory, language, executive function and attention. Thus, perinatal hypoxia has been shown to account for the subsequent profile of long-term cognitive impairment in the general population^45^. For example, one study showed that compared to controls, children with perinatal asphyxia had smaller hippocampal volumes that were associated with poorer long-term visuospatial memory^46^. Another study showed that even mild oxygen deprivation at, or immediately after, birth was related to an increased risk of developmental delays later in childhood: those with mild to moderate acidosis (a measure of risk of hypoxia) displayed significantly lower (p < 0.05) verbal and visuospatial test scores than the low-risk group^47^. Importantly, the group with higher perinatal blood acidity had lower Apgar scores at both 1 min and 5 min compared to the low-risk group. Although profound perinatal events may cause obvious neurological deficit, milder problems may also cause more subtle defects in functioning that are detectable only later as the child develops^35,48^.

Although most follow-up studies examining the long-term prognostic value of Apgar scores on child outcomes have focused on extremely low Apgar scores at 5 min^30,49^, some have examined the developmental correlates across the entire spectrum of recorded Apgar scores (0–10) at 1 and 5 min^31–33^. These latter studies support the hypothesis that even mild degrees of concerns at birth can be associated with long-term cognitive problems. For example, in a study of more than 150,000 children (ages 5–7 years)^32^, lower Apgar scores at 1 and 5 min that were still within the so-called normal range (7–9) were associated with a significant increase in risk of poor developmental outcomes (e.g., language, cognitive development, and general knowledge).

Other studies have reported that infants with low transient Apgar scores at 1 min are at risk for long-term negative developmental consequences even if their scores improve at the 5- and 10-min readings^31,50^. For example, a study of 452 children with Attention-Deficit/Hyperactivity Disorder (ADHD) showed that infants with “poor” (≤ 6) Apgar scores at 1 min had more severe ADHD symptoms later in childhood. This same study reanalyzed the data including only children who had normal Apgar scores ≥ 7) at 5 min, and the results were similar (i.e., low 1-min scores predicted worse outcomes later), suggesting that even transient low scores at 1 min are enough to lead to an increase in ADHD symptomatology. In a similar line, another study on more than 170,000 participants reported that infants who had low Apgar scores at 1 min had higher risk of low IQ scores at age 18, even if these infants achieved a normal (> 7) Apgar score at 5 min^31^.

Although Apgar scores have prognostic value for the general population, there are no comparable data for DS. This is unfortunate because such data would be valuable in (a) suggesting the need for research into the role of specific neonatal characteristics and perinatal events in producing different cognitive outcomes among individuals with DS and (b) highlighting those infants with DS most in need of particular early and intensive behavioral supports and intervention. The present study, therefore, was designed to determine whether Apgar scores at 1 and 5 min in newborn infants with DS are associated with impairments in specific cognitive domains (e.g., attention, memory, executive functioning, and language) previously related to perinatal events in other populations in later childhood, adolescence, and early adulthood, after controlling for important individual, demographic, and environmental factors. We hypothesized that individuals with DS with a lower Apgar score at 1 or 5 min compared to those with higher Apgar scores will exhibit more severe cognitive difficulties later in life.

## Methods

### Participants

The participants were drawn from the Down Syndrome Cognition Project (DSCP), which has generated previous publications^51–53^, although none with the specific aims of the present study. Participants and measures reported here are a subset of those from the larger study. See Ref.^51^ for further details regarding study procedures and data collection.

Participants in the current study were 168 individuals with DS (81 females and 87 males), aged 6 to 25 years at the time of the cognitive evaluation (M = 13.5; SD = 4.8), and for whom English is the primary language spoken at home. Participants were included in the DSCP study if: (1) the trisomy 21 had been verified by karyotype; (2) the biological mother was available for participation; and (3) birth history was available. The participating mother provided written consent, and participants with DS provided verbal or written assent (when capable), before data collection. Participants were excluded from the DSCP study if they had: (1) other chromosomal anomalies; (2) a gestational age(GA) < 35 weeks; (3) > 7 days in NICU only if gestational age was 35–37; (4) > 48 h of oxygen support only if gestational age was 35–37; (5) lack of oxygen at birth greater than 5 min; (6) untreated epilepsy or other seizure disorder; (7) a history of head injury, chemotherapy, or accidental poisoning; (8) untreated severe hearing or vision loss; or (9) an incidence of a loss of consciousness > 5 min. From the initial sample of 338 participants, 29 were excluded from the DSCP study due to the following reasons: > 7 days at NICU only if gestational age was 35–37 (3), lack of oxygen at birth greater than 5 min (5), history of head injury (1), history of chemotherapy (1), the trisomy 21 was not verified by karyotype (4), untreated severe vision loss (1), untreated severe hearing loss (1), untreated epilepsy or other seizure disorder (6), no birth history (2), and gestational age less than 35 weeks (5). Permission to obtain medical records, collection, and abstraction of those records was obtained once families consented. In addition to these 29 excluded participants, 141 participants were excluded from the current study due to not having information from medical records about Apgar scores at 1 and 5 min after birth.

### Measures

The Apgar score at 1 min after birth and the Apgar score at 5 min after birth were abstracted from medical records. The Apgar scoring system is a comprehensive screening tool used to evaluate the newborn’s physical condition^54^. It is given at birth by a clinical health professional who evaluates five variables: heart rate, respiratory effort, muscle tone, reflex irritability, and color. Each element is scored 0, 1, or 2. A total score between 0 and 3 categorizes the baby as severely depressed, from 4 to 6 as moderately depressed and from 7 to 10 as normal. This evaluation is done at 1 min and 5 min after birth in all infants. In general, a 10-min score is required only for infants who score 7 or less at the 5-min Apgar score, and for those requiring resuscitation as a method for monitoring response^37^. For this reason, only 8 participants (1 missing value) had 10-min Apgar scores. Due to the small sample size, we are only able to provide descriptive statistics on these 7 participants (Table 2).

We also obtained socio-demographic information about the participants with DS and their family (e.g., race of the participant with DS, household income, and maternal level of education) via maternal questionnaire.

In addition, participants with DS were administered a set of cognitive measures that targeted the constructs of verbal cognition, fluid reasoning and visual processing, attention, memory and learning, and visual and auditory working memory as measures of executive function. The measures selected have all been used in previously published studies involving children, adolescents, and young adults with DS^10,55,56^.

The Verbal Knowledge and the Riddles subtests from the Kaufman Brief Intelligence Test (KBIT-2)^57^ were used as general measures of verbal cognition. The Verbal Knowledge subtest measures receptive vocabulary. This subtest consists of 60 items. The examiner says a word or asks a question and the participant responds by pointing to the picture that best answers the question. The score is determined by the number of items successfully answered by the participant (items correct), with possible scores ranging from 0 to 60. The Riddles subtest measures verbal comprehension and production. The subtest consists of 48 items. In each item, the examiner says a verbal riddle and the participant responds by pointing to a picture or saying a word that answers the riddle. The score is determined by the number of items answered correctly, with the possible range being 0 to 48.

The Matrices subtest, which is also part of the KBIT-2, was included as a measure of fluid reasoning (involving solving novel problems) and visual processing (involving perceptual, manipulation, and thinking abilities in a visual context)^58^. The subtest consists of 46 multiple-choice items. For the first nine items, the examinee chooses which of five pictures best matches concepts portrayed in the single stimulus picture. For Items 10 to 46, the examinee must choose which of six pictures best completes a matrix. The score is the number of items answered correctly, with possible range being 0 to 46.

The Simple Reaction Time (SRT) subtest from the Cambridge Neuropsychological Test Automated Battery (CANTAB)^59^ was included as a general measure of attention; this test specifically measures speed of response to a single stimulus. The participant must press the button on a press pad as soon as they see a square appearing in the middle of the screen. Intervals between the participant’s response and the onset of the next stimulus are variable during task performance. The median response latency (in milliseconds) was used for the analyses.

The Paired-Associates Learning (PAL) subtest from the CANTAB^59^ was used to assess visual associative memory and learning. In this task, the participant is presented with patterns shown one at a time in different locations around an empty central space on the screen. Next, a single pattern is presented in the center of the screen and the examinee needs to touch where that pattern was shown previously. The number of patterns in each set increases with success at each stage (ranging from 1 pattern to a maximum of 8 patterns), and the participant has up to 10 opportunities per pattern length to touch the correct locations. The participant’s overall success is typically determined by the highest set of patterns located correctly (ranging from 0 to 8). However, we used the first trial memory score due to observed ceiling effects on this outcome. This score corresponds to the number of patterns correctly located summed across all the patterns completed (range 0–26).

The Spatial Span (SSP; forward recall) is also a subtest of the CANTAB and assesses visual working memory. The SSP is a computerized version of the Corsi Block task in which participants are required to copy a sequence of blocks that are displayed one at a time in the same order as they were originally presented. The number of boxes in the sequence increases from 2 boxes at the start level of the test to 9 boxes at the final stage (stage 8). The test is terminated when the participant fails three consecutive trials at any one stage. The participant’s overall success is typically determined by the longest sequence successfully recalled. However, we used the SSP Total Errors (adjusted) for the number of items presented as our primary variable of interest to avoid observed floor effects. The SSP Total Errors (adjusted) is a measure of the participant’s efficiency in attempting the test. Thus, whilst a participant may pass all 8 stages, a substantial number of errors may be made in doing so. Therefore, it is crucial to note that participants failing at any stage of the test have had less opportunity to make errors than participants who complete the test. The SSP Total Errors (adjusted) measure compensates for this difference in opportunity. As stated in the CANTAB manual, the Total Errors (adjusted) is calculated by summing the number of stages not attempted and subtracting the number of stages completed divided by the span length (number of boxes) of the last stage attempted from it. This result is then multiplied by the number of trials allowed per stage (three). For example, for a participant who successfully passed the first two stages with just one trial per stage but did not pass the third stage after the 3 attempts, the score would be: ((5 − (3/4)) × 3 = 12.75, while for a participant who passed the first three stages with just one trial per stage but did not pass the fourth stage after the 3 trials would be: ((4 − (4/5)) × 3 = 9.6. Note that lower scores reflect better performance.

The Recall of Digits Forward is a subtest of the Differential Ability Scales (DAS-II)^60^ and was used to assess auditory working memory. In this task, participants listen to a sequence of random digits read aloud by the examiner and then immediately recall the sequence in the same order as presented. The length of the sequence starts at 2 and increases to a maximum of 10 digits, stopping when the participant can no longer recall the sequences correctly. The score used is the total number of sequences correctly recalled. Scores range from 0 to 38.

We detected the presence of ceiling or floor effects in the cognitive variables by computing frequencies and percentages for each outcome. Measures on which 15% or more of the sample obtained the maximum or the minimum score, and/or exhibited a significant absolute skewness index (> 2), were categorized as having ceiling or floor effects. As previously indicated, for those specific variables showing floor or ceiling effects, we used another variable derived from that same test.

### Statistical analysis

**Figure 1:**
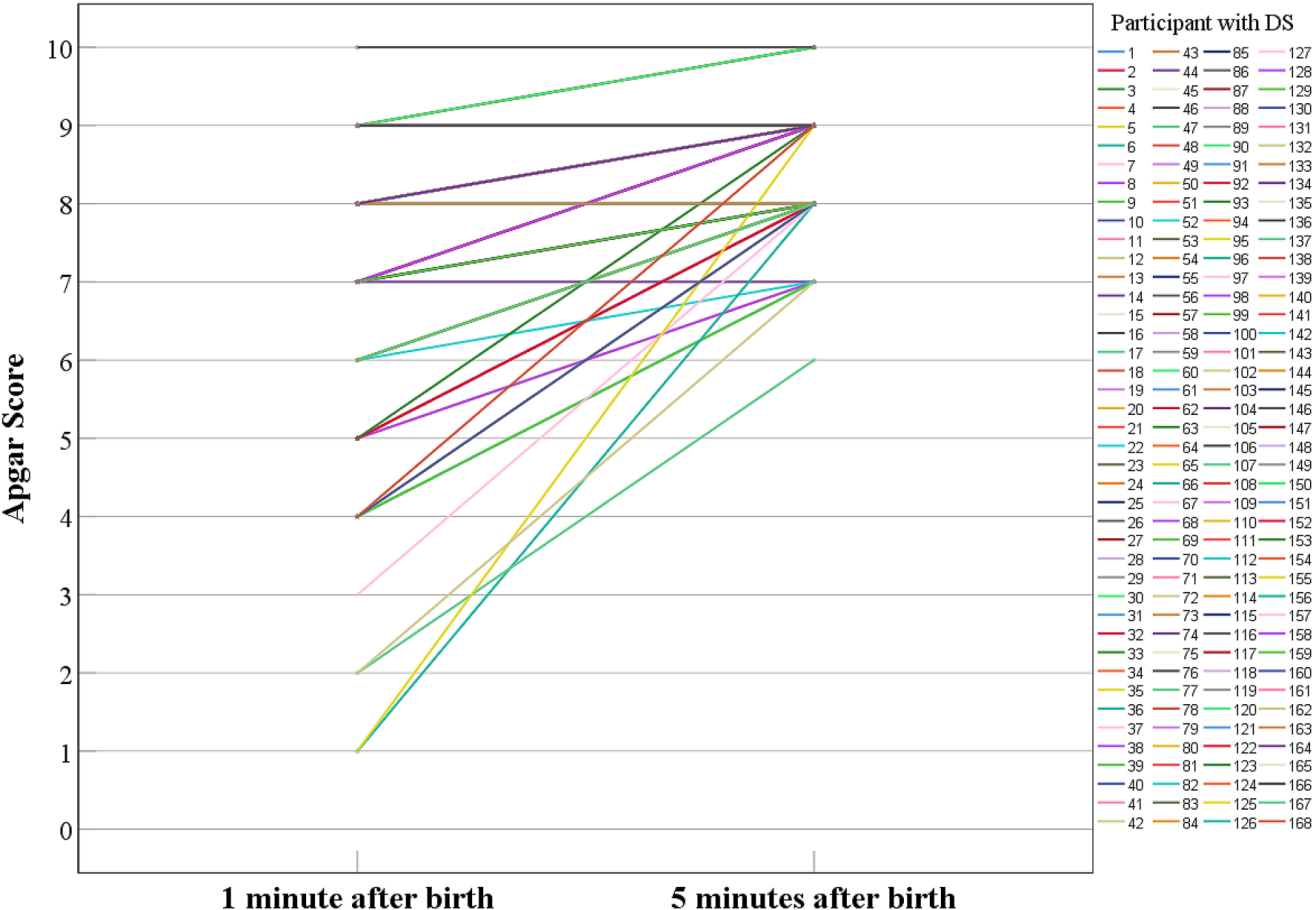
Spaghetti plot representing Apgar score trajectory from one to five minutes after birth in each participant with DS. Each line represents a participant with DS. Several trajectories overlap between them.

The first step was to conduct a descriptive analysis of the sociodemographic and clinical parameters and the cognitive outcomes. Results are described using means, standard deviations, and ranges for numeric variables and absolute and relative frequencies for categorical variables (see Tables 1, 2). In addition, the magnitude of the difference between the Apgar score at 1 and 5 min was calculated with Cohen’s effect size for repeated measures (“Cohen’s *d*”)^61^, along with the 95% confidence interval. We categorized the difference as “large” if effect size differences were greater than 1 pooled standard deviation (|d|> 1)). In addition, the magnitude of change for each participant was graphically represented as a spaghetti plot in Fig. 1. Finally, in order to analyze whether scores at 1 min were significantly related to the Apgar score at 5 min, as well as to the magnitude of change between both scores, we examined the correlation between the Apgar score at 1 min, the Apgar score at 5 min, and the magnitude of the difference between the Apgar score at 1 and 5 min. This association was evaluated using Spearman’s correlation coefficient since data from the Apgar score were not normally distributed in our sample.

**Table 1:** Descriptive analysis of the sociodemographic parameters of the 168 participants with DS and their families.

	AF	RF (%)
**Sex of participant with DS**		
Male	80	47.6
Female	87	51.8
Missing data	1	0.06
**Race of participant with DS**		
African American	14	8.3
Hispanic	3	1.8
Other	22	13.1
Caucasian	128	76.6
Missing data	1	0.06
**Household income**		
$10,000–$25,000	3	1.8
$25,000–$50,000	13	7.7
$50,000–75,000	30	17.9
$75,000–100,000	27	16.1
> $100,000	93	55.4
Missing data	2	0.1
**Maternal education level**		
Less than high school	1	0.6
Completed high school or equivalent	9	5.4
Completed technical school	8	4.8
Completed 1–3 years of college	22	13.1
Bachelor’s degree or 4 years of college	75	44.6
Master’s degree	36	21.4
Doctoral or professional degree	15	8.9
Missing data	2	0.1
**Paternal education level**		
Less than high school	1	0.6
Completed high school or equivalent	14	8.3
Completed technical school	10	6
Completed 1–3 years of college	16	9.5
Bachelor’s degree or 4 years of college	67	39.9
Master’s degree	26	15.5
Doctoral or professional degree	19	11.3
Missing data	14	8.3

First column shows values for absolute frequencies, and second column shows values for relative frequencies.

*DS* Down syndrome, *AF* absolute frequency, *RF* relative frequency.

**Table 2:** Descriptive statistics of clinical and cognitive data of the 168 participants with DS.

	n	Mean	SD	Min–Max	Missing data
**At birth data**					
Maternal age at birth	167	35.21	4.84	22–47	1
Apgar score at 1 min after birth	168	7.45	1.5	1–10	0
Apgar score at 5 min after birth	168	8.65	0.64	6–10	0
Apgar score at 10 min after birth	7	8.71	0.49	8–9	1
**Data collected at time of cognitive testing**					
Chronological age of participant with DS	167	13.54	4.85	6–25	1
SRT (latency in milliseconds)	145	686.29	316.17	242.5–2090.5	23
PAL (first trial memory score)	163	8.29	6.49	0–25	5
SSP forward (total errors adjusted)	146	17.1	4.47	6.8–24	22
Recall of digits forward (total number correct)	135	7.49	4.87	0–25	33
Verbal knowledge (total number correct)	166	14.91	8.31	0–33	2
Riddles (total number correct)	166	9.85	5.71	0–23	2
Matrices (total number correct)	166	12.67	6.36	0–29	2

The first set of measures is related to the data collected at birth for the participants with DS. The second set of measures are those collected at the time of testing (6 to 25 years after birth). Results are described using central tendency (mean values) and variability (standard deviation and range) for numeric variables, and absolute and relative frequencies for categorical variables. Missing values were primarily due to administrative issues, technical issues, or the participant not being compliant.

*n* number of participants, *SD* standard deviation, *min* minimum value reported, *max* maximum value reported, *DS* Down syndrome, *SRT* simple reaction time, *PAL* paired-associates learning, *SSP* spatial span.

For the primary analyses, we first examined the association between Apgar score at 1 and at 5 min and cognitive outcomes (i.e., the median latency in milliseconds from SRT, the first trial memory score from PAL, the total errors adjusted from SSP Forward, and the total number of correct responses for Recall of Digits Forward, Verbal Knowledge, Riddles, and Matrices) with each of the following potential covariates: (1) chronological age (CA) of participant with DS at time of cognitive evaluation, (2) sex of participant with DS, (3) maternal age at birth, (4) maternal level of education, and (5) family income. These associations were evaluated using Spearman’s correlation coefficient for numeric variables not normally distributed, Pearson’s correlation coefficient for numeric variables normally distributed and ANOVA models for categorical variables. Correction for multiple tests was performed using the Bonferroni procedure to maintain a familywise alpha level of p ≤ 0.05.

In the primary analyses, we examined the contribution of the Apgar score to prediction of each cognitive outcome, with the 1- and 5-min scores included in separate simultaneous regression models. The regression models were adjusted, when necessary, for potential covariates (i.e., if a potential covariate was related (p < 0.05) with Apgar scores or cognitive outcomes). This approach led to only CA being added as a covariate in all the models. Predictor variables were added to each regression model simultaneously. See Tables 4 and 5 for a visual representation of each model. Supplementary analyses were conducted to fit models that included both the 1- and 5-min Apgar scores in the same model as well as CA. In the supplementary analyses, we also assessed the indirect effect of the 1-min Apgar score on cognitive outcomes through a possible mediator (i.e., the 5-min Apgar score). Confidence intervals for the indirect effect were constructed based on 5000 bootstrapped samples; if the confidence interval includes zero, there is no evidence of mediation. Because ours is the first analysis on this topic, we provide unadjusted p-values. However, we also indicate which p-values are significant at a familywise p ≤ 0.05 after adjusting for multiple comparisons through the Bonferroni correction procedure (bolded values in tables). Finally, due to a considerable number of missing values for the outcome measures of SSP, PAL and Digits, we conducted sensitivity analyses that entailed parallel regression models with only those participants who had no missing value for any cognitive measure (n = 106). Note that all the cognitive variables included in the models were normally distributed (e.g., skewness (− 1, 1) and (− 2, 2) for kurtosis).All analyses were performed using the statistical software packages SPSS (Version 18.0; SPSS Inc., Chicago, IL, USA), with the exception of the bootstrapped confidence interval for the indirect effect, which were conducted using R (version 4.0.3; The R Foundation for Statistical Computing, Vienna, Austria)^62,63^.

### Ethical approval

This study was approved by the Institutional Review Boards (IRB) of each participating site; Emory University (IRB00005100); University of California, Davis (IRB395392-1); University of Arizona (IRB00001751), Johns Hopkins University (IRB00031164), Oregon Health & Science University (IRB00003602), Children’s National Medical Center (IRB:Pro00002478), University of Wisconsin-Madison (IRB:SE-2010-0016) and University of Illinois Urbana-Champaign (IRB#17424).

The authors assert that all procedures contributing to this work comply with the ethical standards of the relevant national and institutional committees on human experimentation and with the Helsinki Declaration of 1975, as revised in 2008. Informed consent was obtained from the parent or guardian of each participant before testing.

## Results

Sociodemographic characteristics of the sample are presented in Table 1. Descriptive data for the clinical and cognitive measures of interest are presented in Table 2 along with the sample size (and missing values) for each variable. As seen in Fig. 1, the Apgar score significantly improved from 1 to 5 min (95% CI [0.81, 1.62]; df = 168; |d|= 1.8). For example, 25 participants (15% of the sample) had an Apgar score < 7 at 1 min after birth, but only 1 participant (0.05% of the sample) had an Apgar score < 7 at 5 min after birth. All 56 participants with an Apgar score < 8 at 1 min improved their score at 5 min. For those 87 participants with an Apgar score of 8 at 1 min, 14 had the same score at 5 min and 73 improved to 9. Of those 24 participants with an Apgar score of 9 at 1 min, 20 stayed the same and 4 improved. For the 1 participant with an Apgar score of 10 at 1 min, her Apgar score at 5 min stayed the same. The Apgar scores at 1 and 5 min were significantly correlated (r = 0.66; 95% CI [0.6, 0.7]); df = 168; p < 0.001). In addition, Apgar scores at 1 min were negatively correlated with the magnitude of change between the Apgar score at 1 min and 5 min (r =  − 0.81; 95% CI [− 0.9, − 0.7]); df = 168; p < 0.001). Thus, the lower the Apgar score at 1 min, the greater the improvement from 1 to 5 min.

With the exception of CA of the participants at the time of cognitive testing, we found no significant associations between the demographic variables (maternal level of education, maternal age at childbirth, family income, and sex of participants with DS) and either the Apgar scores or any of the cognitive outcomes. As expected, CA of the participant was positively correlated with all cognitive outcomes (Table 3).

**Table 3:** Association between Apgar scores and cognitive outcomes with each of the potential covariates.

	SRT	PAL	SSP	Digits	Verbal knowledge	Riddles	Matrices	Apgar at 1 min	Apgar at 5 min
**Family income**									
F	− 0.12	− 0.05	− 0.04	0.41	0.11	0.09	0.04	0.08	0.08
p-value	0.89	0.53	0.68	0.64	0.16	0.24	0.66	0.33	0.29
95% CI	[− 0.05, 0.01]	[− 0.05, 0,1]	[− 0.05, − 0.01]	[− 0.05, 0.06]	[− 0.05, − 0.01]	[− 0.05, 0.12]	[− 0.05, 0.87]	[− 0.04, 0.21]	[− 0.05. 0.06]
N	144	162	145	134	165	165	166	166	166
**Maternal educational level**									
F	0.78	0.56	0.7	0.34	1.32	2.3	1.4	2	1.69
p-value	0.56	0.73	0.63	0.89	0.27	0.06	0.21	0.05	0.8
95% CI	[− 0.06, 0.05]	[− 0.06, 0.19]	[− 0.06, 0.04]	[− 0.06, 0.05]	[− 0.06, 0.1]	[− 0.06, 0.17]	[− 0.06, 0.11]	[− 0.06, 0.18]	[− 0.06, 0.13]
N	144	162	145	134	165	165	166	166	166
**Maternal age at birth**									
r	0.20	− 0.76	− 0.03	0.04	− 0.12	− 0.13	− 0.08	− 0.06	− 0.12
Sig	0.02	0.34	0.75	0.66	0.13	0.09	0.29	0.42	0.14
95% CI	[− 0.05, 0.39]	[− 0.35, 0.03]	[− 0.28, 0.09]	[− 0.24, 0.2]	[− .0.34, 0.08]	[− 0.34, 0.08]	[− 0.28, 0.09]	[− .015, 0.3]	[− 0.24, 0.2]
N	145	163	146	135	166	166	166	167	167
**Chronological age of participant with DS**									
r	− 0.35	0.22	− 0.29	0.22	0.52	0.41	0.37	− 0.17	− 0.20
p-value	< 0.001***	< 0.01**	< 0.001***	0.01*	< 0.001***	< 0.001***	< 0.001***	0.05	0.13
95% CI	[− 0.49, − 0.01]	[0.04, 0,37]	[− 0.49, − 0.13]	[0.16, 0.29]	[0.31, 0.65]	[0.22, 0.54]	[0.01, 0.49]	[− 0.38, − 0.16]	[− 0.35, 0.1]
N	145	163	146	135	166	166	166	167	167
**Sex of participant with DS**									
F	0.11	0.41	0.19	0.83	1.38	3.15	1.81	0.01	0.09
p-value	0.74	0.53	0.67	0.36	0.24	0.08	0.18	0.93	0.75
95% CI	[− 0.01, 0.02]	[− 0.01, 0.04]	[− 0.01, − 0.01]	[− 0.01, 0.05]	[− 0.01, 0.13]	[− 0.01, 0.19]	[− 0.01, 0.08]	[− 0.01, 0.03]	[− 0.01, 0.04]
N	145	163	146	135	166	166	166	167	167

*r* coefficient of correlation, *F* ANOVA F-value, *95% CI* 95% Confidence Intervals, *N* sample size, *SRT* simple reaction time: median latency in milliseconds, *PAL* paired-associates learning: first trial memory score, *SSP* spatial span Forward: total errors adjusted; Recall of Digits Forward (Digits), Verbal Knowledge, *Riddles and Matrices* total number correct.

Bolded p-values are those which remained significant after correcting for multiple comparisons (p < 0.001).

**Figure 2:**
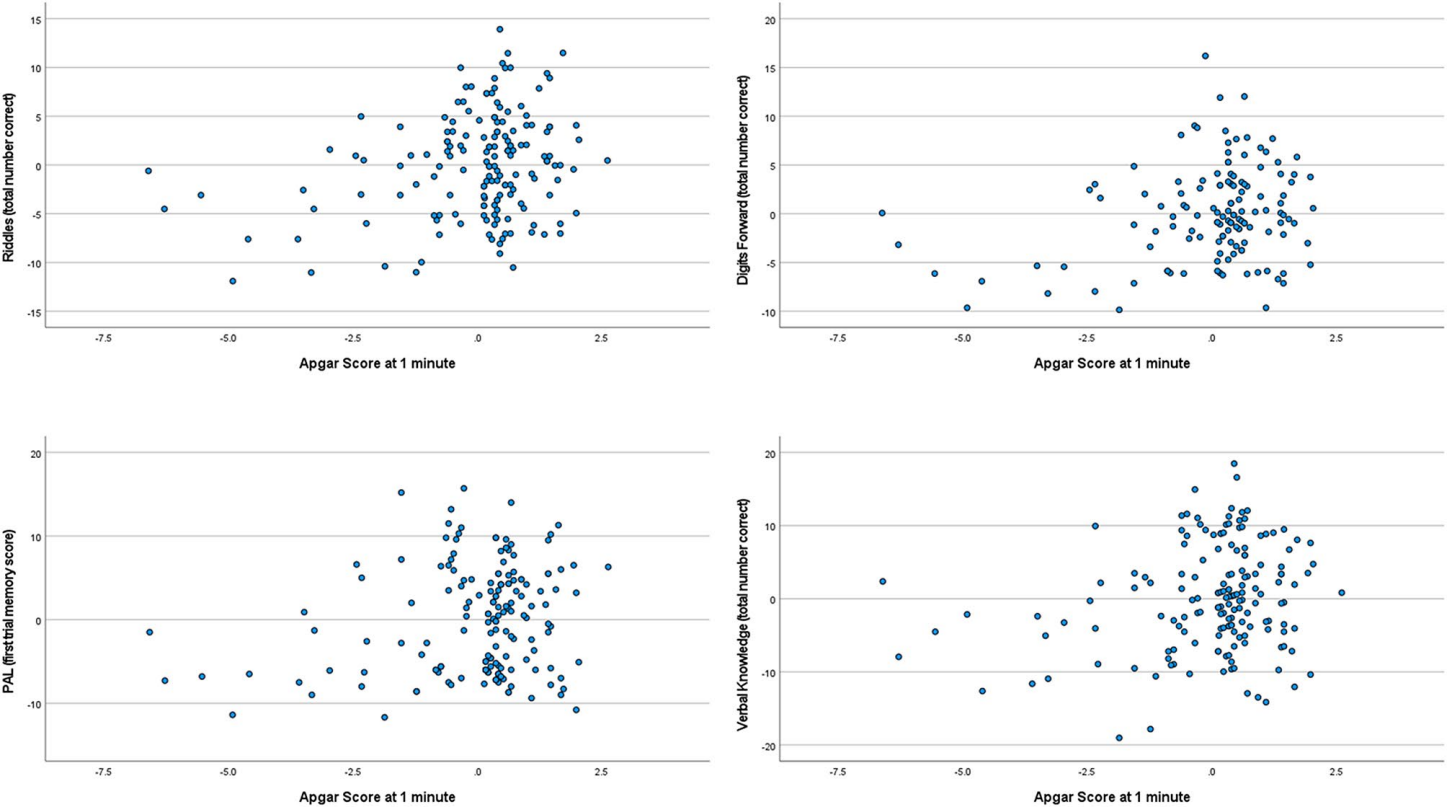
Partial regression plots representing the association between the Apgar score at 1 min (x-axis) and the following dependent variables (y-axis) from the top left to the bottom right: Riddles, Recall of Digits Forward, PAL(first trial memory score) and Verbal Knowledge, while adjusting for chronological age (CA) at time of cognitive testing. See that the score on cognitive variables (Y) increases as Apgar score (X_1_) increases adjusting for chronological age (X2). *PAL* paired-associates learning.

**Figure 3:**
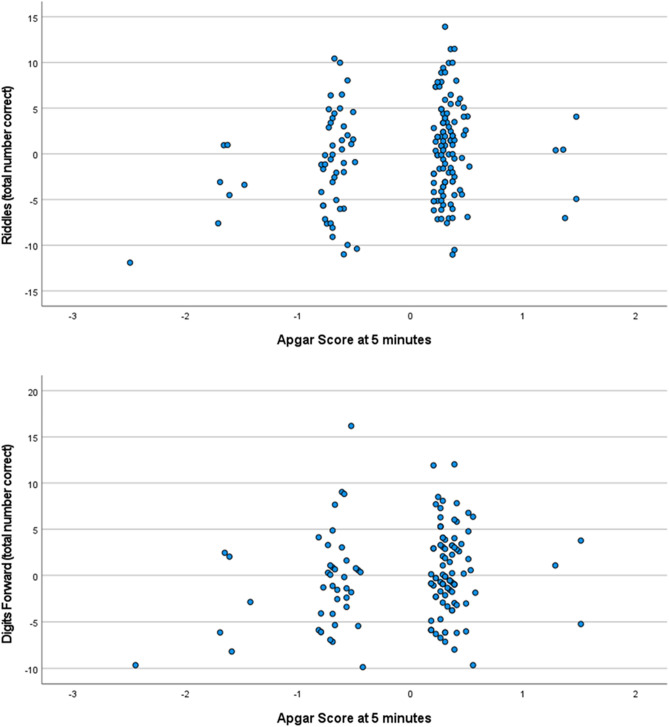
Partial regression plots representing the association between the Apgar score at 5 min (x-axis) and the following dependent variables (y-axis): Riddles and Recall of Digits Forward (total correct), while adjusting for chronological age (CA) at time of cognitive testing. See that the score on cognitive variables (Y) increases as Apgar score (X_1_) increases adjusting for chronological age (X2).

In the primary analyses, the linear regression models showed that the Apgar score at 1 min predicted the number of correct items on the Verbal Knowledge (β = 0.99; df = 163; p < 0.01) and the Riddles (β = 0.24; df = 163; p = 0.001) subtests of the KBIT-2, the number of patterns correctly located after the first trial on the PAL subtest of the CANTAB (β = 0.25; df = 160; p < 0.01), the number of adjusted mistakes on the SSP subtest of the CANTAB (β = -− 0.20; df = 143; p = 0.014) and the total number of sequences correctly recalled on the Recall of Digits Forward subtest of the DAS-II (β = 0.25; df = 132; p < 0.01). See Table 4 for the results for the regression models for the primary analyses and Fig. 2 for an illustration of the findings. In all cases, a higher Apgar score was associated with better performance on the cognitive outcome. The Apgar score at 1 min, however, did not predict the level of performance in the remaining cognitive assessments (SRT and the Matrices subtest). The Apgar score at 5 min predicted only the number of correct items on the Riddles subtest of the KBIT-2 (β = 0.17; df = 163; p = 0.02) and the total number of sequences correctly recalled on the Recall of Digits Forward subtest of the DAS-II (β = 0.17; df = 132; p = 0.05); again, a higher Apgar score predicted a better score on the cognitive outcomes (see Table 5 for the regression results and Fig. 3 for an illustration of the findings).

**Table 4:** Apgar score at 1 min associated with cognitive outcomes while adjusting for chronological age at time of cognitive testing: results from regression analyses.

Explanatory variable	N	β	Partial R^2^	df	95% Confidence interval	p-value	Dependent variable
Apgar Score at 1 min	145	− 0.11	− 0.11	142	[− 60.70, 11.72]	0.18	SRT (latency)
Chronological age		− 0.38	− 0.37		[− 36.29, − 14.87]	** < 0.001**	
Apgar Score at 1 min	163	0.25	0.19	160	[0.16, 1.47]	** < 0.01**	PAL (first trial memory score)
Chronological age		0.30	0.25		[0.14, 0.54]	** < 0.001**	
Apgar Score at 1 min	146	− 0.20	− 0.20	143	[− 1.07, − 1.24]	0.01	SSP forward (total errors adjusted)
Chronological age		− 0.32	− 0.32		[− 0.45, − 0.16]	** < 0.001**	
Apgar Score at 1 min Chronological age	135	0.25	0.26	132	[0.28, 1.32]	** < 0.01**	Digits forward (total number correct)
Chronological age		0.26			[0.09, 0.41]	** < 0.01**	
Apgar Score at 1 min	166	0.99	0.21	163	[0.26, 1.72]	< 0.01	Verbal knowledge (total number correct)
Chronological age		0.94	0.54		[0.71, 1.17]	** < 0.001**	
Apgar Score at 1 min Chronological age	166	0.24	0.26	163	[0.37, 1.43]	** < 0.01**	Riddles (total number correct)
Chronological age		0.45	0.45		[0.07, 0.70]	** < 0.001**	
Apgar Score at 1 min	166	0.13	0.14	163	[− 0.06, 1.17]	0.08	Matrices (total number correct)
Chronological age		0.39	0.39		[0.33, 0.71]	** < 0.001**	

*N* sample size, *R* coefficient of correlation, *df* degrees of freedom, *SRT* simple reaction time, *PAL* paired-associates learning, *SSP* spatial Span, *Digits Forward* recall of digits forward.

Bolded p-values are those which remained significant after Bonferroni correction (p ≤ 0.007) for multiple comparisons. Confidence interval corresponds to the unstandardized coefficient.

**Table 5:** Apgar score at 5 min associated with cognitive outcomes while adjusting for chronological age at time of cognitive testing: results from regression analyses.

Explanatory variable	N	β	Partial R^2^	df	95% Confidence interval	p-value	Dependent variable
Apgar Score at 5 min	145	− 0.12	− 0.13	142	[− 141.97, 16.24]	0.12	SRT (latency)
Chronological age		− 0.38	− 0.37		[− 35.64, − 14.7]	< 0.01	
Apgar Score at 5 min	163	0.05	0.05	160	[− 1.05, 2.1]	0.51	PAL (first trial memory score)
Chronological age		0.23	0.23		[.1, .51]	** < 0.01**	
Apgar Score at 5 min	146	− 0.07	− 0.08	143	[− 1.66, 0.62]	0.37	SSP forward (total errors adjusted)
Chronological age		− 0.30	− 0.30		[− 0.43, − 0.13]	** < 0.001**	
Apgar Score at 5 min	135	0.17	0.17	132	[0.01, 2.6]	< 0.05	Digits forward (total number correct)
Chronological age		0.25	0.24		[0.08, 0.40]	** < 0.01**	
Apgar Score at 5 min	166	0.12	0.14	163	[− 0.14, 3.37]	0.07	Verbal knowledge (total number correct)
Chronological age		0.53	0.53		[0.69, 1.14]	** < 0.001**	
Apgar Score at 5 min	166	0.17	0.19	163	[0.31, 2.86]	0.02*	Riddles (total number correct)
Chronological age		0.43	.43		[0.35, 0.68]	** < 0.001**	
Apgar Score at 5 min	166	0.14	0.14	163	[− .06, 1.17]	0.06	Matrices (total number correct)
Chronological age		0.39	0.39		[0.33, 0.71]	** < 0.001**	

*N* sample size, *R* coefficient of correlation, *df* degrees of freedom, *SRT* simple reaction time, *PAL* paired-associates learning, *SSP* spatial Span, *Digits Forward* recall of digits forward.

Bolded p-values are those which remained significant after Bonferroni correction (p ≤ 0.007) for multiple comparisons. Confidence interval corresponds to the unstandardized coefficient.

Similar results were found in the sensitivity analyses in which only those participants who completed all the cognitive tasks were included. In particular, the Apgar score at 1 min predicted the following outcome measures while adjusting for CA: Verbal Knowledge (β = 0.20; df = 105; p = 0.01) and the Riddles (β = 0.26; df = 105; p = 0.005) subtests of the KBIT-2, the number of patterns correctly located after the first trial on the PAL subtest of the CANTAB (β = 0.22; df = 105; p = 0.02), the number of adjusted mistakes on the SSP subtest of the CANTAB (β =  − 0.27; df = 105; p < 0.01), and the total number of sequences correctly recalled on the Recall of Digits-Forward subtest of the DAS-II (β = 0.31; df = 105; p = 0.001). In addition, the sensitivity analyses showed that the Apgar score at 5 min predicted only the number of correct items on the Matrices subtest of the KBIT-2 (β = 0.19; df = 105; p = 0.05) and the total number of sequences correctly recalled on the Recall of Digits-Forward subtest of the DAS-II (β = 0.19; df = 105; p = 0.05).

In the supplementary analyses, we fit models that included both the 1- and 5-min Apgar scores in the same model (see Supplementary Table S1). Due to their high correlation and thus collinearity, results of these models should be interpreted with caution. In general, the magnitude of the coefficients for the 1-min Apgar score were reduced relative to the primary analyses, although still statistically significant assuming an unadjusted p-value < 0.05. The magnitude of the coefficients for the 5-min Apgar score also were reduced relative to the primary analyses, but lost significance for the few outcomes for which it was significant in the primary analysis. A further evaluation of the 5-min Apgar score as a possible mediator in the model found insufficient evidence of the mediation (Supplementary Table S1).

## Discussion

The current study was designed to determine whether Apgar scores at 1 and 5 min post-delivery in newborn infants with DS are associated with cognitive functioning in later childhood, adolescence, and early adulthood (after controlling for relevant sociodemographic variables).

We found that the Apgar score at 1 min predicted long-term cognitive outcomes related to receptive vocabulary, verbal comprehension and production, verbal and visual working memory, and visual memory and learning, but not outcomes reflecting visual attention, or fluid reasoning and visual processing. Previous research has suggested that a low Apgar score at 1 min may reflect acute events compromising oxygen availability during the birth process^38,64^. Studies conducted in the euploid population suggest that, in babies born prematurely, even minor bouts of hypoxia at birth may be associated with damage to periventricular white matter^47^. In addition, neonatal white matter abnormality has been shown to be an important predictor of later abilities related to producing, understanding, and synthesizing speech and language in otherwise typically developing children at age 7^65^. Although our results are consistent with these findings of potential hypoxia-induced white matter damage, it is important to recognize that an Apgar score at 1 min might reflect problems other than hypoxia or respiratory distress^66^. In addition, it is important to note that one of the exclusion criteria of the DSCP was suffering from lack of oxygen at birth for more than 5 consecutive minutes. Thus, our results suggest the need for future research to confirm the causal pathways to cognitive impairment for which the 1-min Apgar score might be a proxy.

Most of our results reflecting the link between greater long-term cognitive difficulties and a lower Apgar score are related to the Apgar score at 1 min rather than at 5 min. In fact, the Apgar score at 5 min only predicted long-term cognitive outcomes related to verbal comprehension, verbal production, and verbal working memory. In interpreting this finding, it is important to point out that nearly all of our participants showed considerable improvement in their Apgar scores over the first 5 min after birth. Moreover, there was more variability in the 1-min Apgar score than in the 5-min score; thus, there may have been improved ability to detect an association in the former score. At the same time, however, these results are consistent with findings from the euploid population in that, despite being highly correlated^40,44^, the 1- and 5-min Apgar scores are not reflective of identical risk factors and thus, can have different developmental consequences^39,41–43^.

The fact that a lower Apgar score at 1 min was related to a greater improvement in the score at 5 min suggests that even transient low Apgar scores at 1 min predict long-term cognitive difficulties in individuals with DS. These results, again, are in line with some of the previous studies in the euploid population showing that improvement in the Apgar score from 1 to 5 min immediately after birth is still associated with developmental vulnerability^31,32^. However, these studies had sample sizes large enough to do an analysis for each group that had a specific Apgar score at 1 min. Thus, we would need a higher number of participants with compromised Apgar scores at 1 and 5 min to confirm that hypothesis.

Nonetheless, it is important to point out that although most of our participants were in the “normal” range of Apgar scores (i.e., 7 to 10) at both 1 min (85.2%) and 5 min (99.4%), a lower Apgar score was still associated with greater long-term cognitive difficulties. This finding, too, is in line with some previous results in the euploid population^32^, suggesting that developmental adversity may be best understood as a linear function across the full range of scores. This observation is important because both research and clinical practice generally emphasize the increased risks of adverse outcomes associated with “below normality” (i.e., < 7) Apgar score, generally disregarding those babies within the normal range (i.e., 7–10).

Finally, the observed associations between cognitive outcomes and the Apgar score at 1 min and 5 min were more pervasive in the verbal domains of cognition. These results could be due to the specific cognitive outcome measures selected for the constructs of interest rather than the constructs themselves, thereby reflecting methodological differences in the measures. However, the fact that literature indicates that verbal skills usually lag behind nonverbal cognitive skills for individuals with DS^67,68^ suggests that there should be a neurodevelopmental explanation for that trend in our results; language ability is one of the most impaired skills across their lifespan^69^. Thus, brain areas associated with verbal skills may be the most vulnerable to other forms of injury and insult in DS. Whether this enhanced vulnerability to early insult is specific to DS, however, will require comparable data on other ID-related conditions. In addition, it is important to note that similar results were found when including only those participants who completed all the cognitive tasks and had no missing data values; therefore, our results suggest that the observed differences in the regression models are not due to the characteristics of the participants who completed or failed to complete all the cognitive measures.

### Limitations

Participants in the present study are part of a larger study (DSCP) in which GA < 35, lack of oxygen at birth for more than 5 min and other perinatal events were exclusion criterions. Including participants, for example, with GA < 35 would have increased the likelihood of having a broader range of Apgar scores at both 1 and 5 min and probably more participants with Apgar scores at 10 min. Thus, our results may underestimate the full impact of perinatal events and risk factors on cognition in individuals with DS.

## Conclusion

In summary, our study shows that even transient low Apgar scores at 1 min (not necessarily within the abnormal range of < 7) are associated with lower cognitive functioning in concrete cognitive areas at 6 to 25 years of age in individuals with DS. These results provide clinicians with valuable prognostic information and the justification to carefully monitor infants with DS who show even mildly compromised Apgar score at 1 and 5 min. However, the Agar scores account for only a small proportion of variance in the cognitive outcomes. Thus, more research is needed to determine: (1) whether the contribution of the 1- and 5-min Apgar score to later cognitive variability is clinically meaningful and (2) how many other factors, including those relevant long after the perinatal period, are important. Moreover, the Apgar score is a proxy for a number of risk factors and adverse events (e.g., oxygen availability at birth, delivery method, passive immunity, gestational age, among others) which will need to be identified in future research.

## Supplementary Information

Supplementary Information.

## Data Availability

The datasets used and/or analyzed during the current study are available from the corresponding author upon reasonable request.
