# Supplementary material for: Relationship between Apgar scores and long-term cognitive outcomes in individuals with Down syndrome
Source: Sci Rep. 2021 Jun 16;11:12707. doi: 10.1038/s41598-021-90651-3 (PMC8208985; doi:10.1038/s41598-021-90651-3)
Supplement: Supplementary file 1 — Supplementary Information. [file 41598_2021_90651_MOESM1_ESM.docx]

**Supplementary Table 1.** Independent stepwise regression analyses for each cognitive outcome (dependent variable) including the Apgar Score at 1 minute, the Apgar Score at 5 minutes and chronological age at time of cognitive testing as independent variables.

| Explanatory variable | N | β | Partial R² | df | 95% Confidence interval | p-value | Mediation effect 95% CI | Dependent Variable |
| --- | --- | --- | --- | --- | --- | --- | --- | --- |
| Apgar Score at 1 minute  Apgar Score at 5 minutes  Chronological age | 145 | -.05  -.10  -.39 | -.04  -.08  -.37 | 141 | [-56.78, 34.47]  [-147.8, 51.92]  [-36.41, -15.98] | .63  .34  **<.001** | [-63.6, 16.2] | SRT  (latency) |
| Apgar Score at 1 minute  Apgar Score at 5 minutes  Chronological age | 163 | .25  -.10  .25 | .20  -.08  .25 | 159 | [.26, 1.91]  [-3.02, .90]  [.13, .54] | .01  .29  **<.01** | [-0.88, 0.21] | PAL  (first trial memory score) |
| Apgar Score at 1 minute  Apgar Score at 5 minutes  Chronological age | 146 | -.24  .07  -.32 | -.20  .06  -.32 | 142 | [-1.31, -.13]  [-.91, 1.89]  [-.449, -.152] | .017  .49  **<.001** | [-0.29, 0.51] | SSP Forward  (total errors adjusted) |
| Apgar Score at 1 minute  Apgar Score at 5 minutes  Chronological age | 135 | .24  .02  .26 | .19  .02  .26 | 131 | [.096, 1.43]  [-1.51, 1.79]  [.09, .41] | .03  .86  **<.01** | [-0.47, 0.44] | Digits Forward  (total number correct) |
| Apgar Score at 1 minute  Apgar Score at 5 minutes  Chronological age | 166 | .17  .02  .55 | .15  .02  .54 | 162 | [-.01, 1.85]  [-1.93, 2.48]  [.71, 1.17] | .05  .81  **<.001** | [-0.49, 0.68] | Verbal Knowledge  (total number correct) |
| Apgar Score at 1 minute  Apgar Score at 5 minutes  Chronological age | 166 | .21  .05  .45 | .18  .04  .45 | 162 | [.12, 1.46]  [-1.16, 2.02]  [.37, .70] | .021  .60  **<.001** | [-0.31, 0.57] | Riddles  (total number correct) |
| Apgar Score at 1 minute  Apgar Score at 5 minutes  Chronological age | 166 | .08  .09  .40 | .06  .09  .37 | 162 | [-.45, 1.10]  [-.95, 2.75]  [.33, .71] | .41  .34  **<.001** | [-0.32, 0.86] | Matrices  (total number correct) |

Notes: Bolded p-values are those which remained significant after Bonferroni correction (p<0.007) for multiple comparisons. Confidence interval corresponds to the unstandardized coefficient. The mediation effect 95% confidence interval is the confidence interval for the indirect effect of the Apgar score at 1 minute on the dependent variable, through the possible mediator (Apgar at 5 minutes) based on 5000 bootstrapped samples. If this confidence interval includes zero, there is no mediation. *Abbreviations:* N, sample size; R, coefficient of correlation; df, degrees of freedom; SRT, simple reaction time; PAL, paired-associates learning; SSP, Spatial Span; Digits Forward, Recall of Digits Forward.
